# Comprehensive Review on Betulin as a Potent Anticancer Agent

**DOI:** 10.1155/2015/584189

**Published:** 2015-03-19

**Authors:** Sylwia Katarzyna Król, Michał Kiełbus, Adolfo Rivero-Müller, Andrzej Stepulak

**Affiliations:** ^1^The Chair and Department of Biochemistry and Molecular Biology, Medical University, 20-093 Lublin, Poland; ^2^Department of Physiology, Institute of Biomedicine, University of Turku, 20520 Turku, Finland; ^3^Faculty of Natural Sciences and Technology, Åbo Akademi University, 20500 Turku, Finland; ^4^Department of Otolaryngology, MSW Hospital, 20-331 Lublin, Poland

## Abstract

Numerous plant-derived substances, and their derivatives, are effective antitumour and chemopreventive agents. Yet, there are also a plethora of tumour types that do not respond, or become resistant, to these natural substances. This requires the discovery of new active compounds. Betulin (BE) is a pentacyclic triterpene and secondary metabolite of plants abundantly found in the outer bark of the birch tree *Betulaceae* sp. BE displays a broad spectrum of biological and pharmacological properties, among which the anticancer and chemopreventive activity attract most of the attention. In this vein, BE and its natural and synthetic derivatives act specifically on cancer cells with low cytotoxicity towards normal cells. Although the antineoplastic mechanism of action of BE is not well understood yet, several interesting aspects of BE's interactions are coming to light. This review will summarize the anticancer and chemopreventive potential of BE *in vitro* and *in vivo* by carefully dissecting and comparing the doses and tumour lines used in previous studies, as well as focusing on mechanisms underlying its activity at cellular and molecular level, and discuss future prospects.

## 1. Introduction

Epidemiological data indicated an increase in the cancer incidence and mortality. According to the GLOBOCAN 2008 estimations, there have approximately been 12.7 million new cancer cases diagnosed and 7.6 million deaths worldwide in 2008 [1]. Furthermore, it has been also prognosed that cancer will exceed heart diseases as the leading cause of death in the world, entailing serious social and economic consequences [2]. Despite the significant development of new surgical techniques, radio-, chemo-, and targeted therapy, failures in tumour treatment are still the most important challenges to oncology [3]. The current radio- and chemotherapy procedures also result in the damage of normal cells and consequently cause a number of serious side effects. Additionally, the acquired drug resistance by tumour cells is considered to be responsible for the failure of conventional types of oncological therapy, including cytostatic drugs and radiation [4]. A novel approach to the cancer treatment has appreciated the key components of specifically altered signalling pathways in neoplastic cells or targeting of the tumour microenvironment without affecting noncancerous cells.

The use of natural plant-derived compounds has been considered to be an interesting aspect for the treatment of human neoplastic diseases. Natural plant-derived substances, relatively easily available due to their commonly occurrence in the nature, seem to constitute a promising group of anticancer or chemopreventive agents and have played a key role in the development of drugs or supplements for the treatment of several human cancers. Of all commercially offered anticancer drugs between 1981 and 2006, no more than 22.2% of the total number have been categorized as synthetic ones [5–7].

The most applicable anticancer drugs derived from plants being in clinical use are taxanes (including paclitaxel isolated from* Taxus brevifolia* Nutt.,* Taxaceae*) [8] and vinca alkaloids (*Catharanthus* alkaloids) (including vinblastine and vincristine, isolated from* Catharanthus roseus* (L.) G. Don,* Apocynaceae*) [9]. Moreover, many derivatives of these substances have been synthesized.

**Figure 1:**
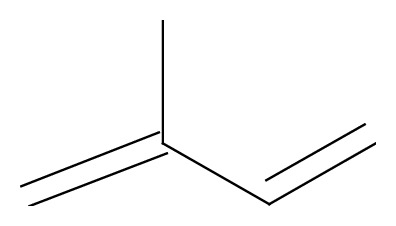
Chemical structure of isoprene.

Terpenes are a large group of widespread secondary metabolites of plants and are considered as potentially useful in cancer pharmacotherapy, because of their selective cytotoxicity towards numerous human cancer cells, as demonstrated* in vitro* and* in vivo* studies. Triterpenes, one of terpenes classes, are formed from six isoprene units (Figure 1) and occur as complex cyclic structures called triterpenoids [10].

**Figure 2:**
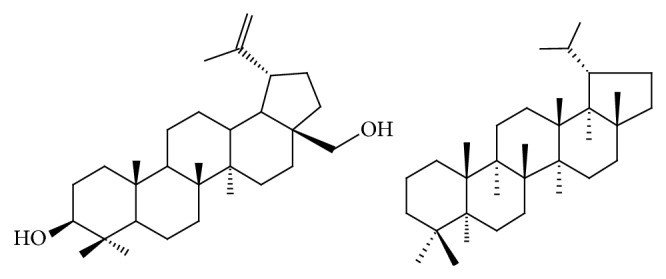
Chemical structure of betulin and lupane.

Betulin (BE, 3-lup-20(29)-ene-3*β*,28-diol), also known as betulinol, betuline, or betulinic alcohol [11], is a pentacyclic lupane-type triterpenoid (Figure 2) naturally distributed in many plants [12, 13]. BE was one of the first natural substances isolated from plants, by Lowitz in 1788, and its chemical structure was finally determined in 1952. Later, BE has been found in other plant species of the* Betulaceae* family, as a component of the outer bark of the birch species,* Betula alba*,* B. pendula*,* B. pubescent,* and* B. platyphylla*. BE has also been found in* Diospyros leucomelas*,* Zizyphus mauritiana*,* Nelumbo nucifera*, seeds of* Ziziphus vulgaris* var.* spinosus*, and in the bark of* Trochodendron aralioides*. BE is predominantly found in a content between 10 and 30% [14], through 34% of dry weight of bark from white birch [15] or even over 50% in the birch bark extract from* B. pendula* Roth [16] and yellow birch (*B. alleghaniensis Britton*) in the Quebec region in Canada [17]. Chemical composition of the birch bark extracts is strongly linked to preparation and purification methods used and influences the percentage of BE which can vary from 54% to 82% of dry weight [16].

Numerous studies have demonstrated that BE elicits a broad range of biological and pharmacological properties, including antibacterial, antifungal, and antiviral activities. However, the anticancer and chemopreventive potential of BE are the focus of most attention [11].

## 2. BE Inhibits Proliferation and Invasion of Different Types of Cancer

BE has been shown to elicit anticancer properties by inhibiting cancer cells growth. Cytotoxicity and antiproliferative potential of BE have been studied in several established cancer cell lines, as well as primary tumour cell cultures (Tables 1 and 2 and references therein) and cancer xenograft models.

Furthermore, there are some data reporting antitumour potential of tropical plants-derived BE, suggesting that* Betulaceae* species may not be the only origin of biologically and pharmacologically active BE. It is considered that BE elicits antiproliferative and cytotoxic activity irrespectively of the natural source. BE isolated from* Chaenomeles sinensis* KOEHNE has had an inhibitory effect (with the IC_50_ 20.9 *μ*M) on soft agar colony formation induced by TPA (12-*O*-tetradecanoyl phorbol-13 acetate) in mouse epidermal cells (JB6 Cl 22, Cl 41 cells) [18], whereas BE from the twigs of* Celtis philippinensis *inhibited proliferation of lung cancer cells [19], and BE from the roots of* Belamcanda chinensis* (L.) DC was effective against breast, prostate, and stomach cancer cells [20]. Likewise, BE from the twigs of* Coussarea paniculata* decreased proliferation of human ovarian carcinoma cells [21], whereas BE from* Cyrtomium fortumei* (J.) inhibited growth of human prostate and stomach cancer cell lines [22].

BE has exhibited quite a different range of its antiproliferative activity, depending on cancer cells type, from a weak inhibition of cell proliferation in human erythroleukaemia cell line (K562) to a strong inhibition in human neuroblastoma cells (SK-N-AS), where the effect has been most pronounced (Table 1 and references therein). Additionally, BE has also been found to express significant cytotoxicity against primary cancer cells cultures isolated from tumour samples obtained from ovarian, cervical carcinoma, and glioblastoma patients, where the IC_50_ values have ranged from 2.8 to 3.4 *μ*M [23] (Table 2), being significantly lower, when compared with established cell lines [21, 24].

Other studies have shown clearly pronounced effect of BE towards human neural tumour cell lines with the IC_50_ value 10.3 *μ*M in TE671 (rhabdomyosarcoma/medulloblastoma), neuroblastoma cells—2.5 *μ*M in SK-N-AS [23], 17.1 *μ*M in GOTO, and 16.5 *μ*M in NB-1 cell line [25], and glial tumour—5.9 *μ*M in C6 (rat glioma) [23].

It should be mentioned that BE has also elicited significant antiproliferative potential against human thyroid carcinoma FTC 238 cells and the concentration 6.8 *μ*M has effectively inhibited proliferation of 50% cells after 48 h treatment [23].

BE has been investigated for its anticancer potential in human lung cancer cells Lu1 (with the IC_50_ values >45.2 *μ*M) [19], NCI-H460 (nonsmall cell lung carcinoma, the IC_50_ value 63.5 *μ*M) [26], and A549. Interestingly, A549 cell line has been extensively studied by several authors, and the IC_50_ values have prominently ranged from 3.8 *μ*M [27, 28] through 7.4 *μ*M [23] and 20 *μ*M [29] to 33.4 *μ*M [26]. Another study has shown that the dose of BE required to reach a 10% cell viability inhibition (ID_10_) in A549 cells has been 0.7 *μ*M and the effect obtained after 24 h has been nearly doubled, when the treatment has been extended to 48 h (0.4 *μ*M) [30]. Moreover, BE has also been found to be slightly more potent antitumour agent than cisplatin (IC_50_ value 25 *μ*M) towards human lung cancer A549 cell line [29], however, was also demonstrated to be inactive towards nonsmall-cell bronchopulmonary carcinoma (NSCLC-N6) [31].

BE has also been evaluated* in vitro* for its anticancer potential towards the most commonly diagnosed cancers in women worldwide [1]. Its inhibitory effect on the growth of human breast, cervical, and ovarian carcinoma cells has been shown. Cell proliferation was 53.2% inhibited by 20 *μ*M of BE in MCF-7 and Bcap-37 cell lines (both breast cancer cell lines) [20]. Other studies have shown that BE at the concentration 10 *μ*M (4.43 *μ*g/mL) and 30 *μ*M (13.28 *μ*g/mL) inhibited 25.81% and 35.54% proliferation of MCF-7 cells, respectively [16], whereas another report has shown the IC_50_ value—8.32 *μ*M [32]. Significantly higher IC_50_ values for MCF-7 cells have been reported in several other studies—23.3 *μ*M [27, 28] and 30.7 *μ*M [26]. T47D cell line has varied significantly in the sensitivity to the antiproliferative properties of BE with the IC_50_ value from 5.2 *μ*M [23] to 73.2 *μ*M [33]. On the other side, BE has been shown to elicit about three-fold weaker antiproliferative activity (IC_50_ value 17 *μ*M) with respect to cytostatic agent 5-fluorouracil (5-FU, with the IC_50_ value 5.34 *μ*M) against MCF-7 cell line [34]. The proliferation of human cervical cancer cells (HeLa cell line) has been inhibited in dose- and time-dependent manner. The IC_50_ values after 24 h were 74.1 *μ*M [24], after 48 h 22.6 *μ*M [26] and 57.1 *μ*M [24], and 6.67 *μ*M [32] and 34.4 *μ*M [24] after 72 h. The dose required to reach an ID_10_ in HeLa has been 0.47 *μ*M, and the effect obtained after 24 h has been significantly enhanced when the treatment has been extended to 48 h (0.32 *μ*M) [30]. Other authors have reported BE to inhibit proliferation of HeLa cells at the concentration 10 *μ*M (4.43 *μ*g/mL) and 30 *μ*M (13.28 *μ*g/mL) by 73.02% and 81.39%, respectively [16]. BE at the concentration >45.2 *μ*M has been demonstrated to reach a 50% cell proliferation inhibition in human ovarian carcinoma cells (A2780 cell line) [21].

Furthermore, some studies have also provided evidence that BE elicits antiproliferative activity towards human prostate cancers including androgen-dependent type. However, high discrepancies appear when comparing the IC_50_ values towards the same cell line PC-3, ranging from 17.9 *μ*M [27, 28] through 82.9 *μ*M [26] up to >250 *μ*M [35]. For example, BE inhibited proliferation of PC-3 cells by 18.4% [22] and by 17.3% at concentration 20 *μ*M [20], whereas in LNCaP cells (androgen-dependent human prostate cancer cell line) the IC_50_ was over 45.2 *μ*M [19].

BE has also been shown to display antiproliferative activity towards cancers within human digestive system. BE has inhibited proliferation by 50% in pancreatic carcinoma (EPP85-181) and human gastric (EPG85-257) cell lines at 21.09 *μ*M and 18.74 *μ*M concentration, respectively [36]. The proliferation of another stomach cancer cell line (MGC-803) was inhibited by 43.7% [20] and 45.1% [22] at a concentration of 20 *μ*M. BE has been investigated for its antiproliferative potential towards human colorectal adenocarcinomas, DLD-1, HT-29, Col2, and SW707 cells. Inhibition of cells proliferation in response to BE has been highly dependent on the cell line. The BE IC_50_ values for DLD-1 [27, 28] and HT-29 colon cancer cells [23] have been comparable, 6.6 *μ*M and 4.3 *μ*M, respectively, and considerably much lower than for Col2 cells, with the IC_50_ values of 45.2 *μ*M [19], and for SW707 cells—51.7 *μ*M [33]. Conversely, BE is ineffective against HT-29 cells, with an IC_50_ value higher than 250 *μ*M [35].

BE has also demonstrated extremely diverse antiproliferative effects on human hepatoma cell lines. The IC_50_ values have ranged from 22.8 *μ*M in HepG2 cells to 132.1 *μ*M in SK-HEP-1 cells [26]. The BE dose required to reach an ID_10_ in HepG2 has been 1.02 *μ*M, and the antiproliferative effect obtained after 24 h has been almost doubled after the treatment time has been extended to 48 h (0.5 *μ*M) [30].

Moreover, BE has been tested with promising results for its cytotoxicity and inhibitory activity towards a series of melanoma cell lines. The BE IC_50_ values in human melanoma cells G361 and SK-MEL-28 have been comparable, 12.4 *μ*M and 16.2 *μ*M, respectively [25], similar to those for murine melanoma B16-F1 cells—13.8 *μ*M [27], but considerably lower than in the case of B16 2F2 [37] and MEL-2 [38] cell lines, suggesting that antiproliferative potential of BE was independent from the cells origin (of human or non-human origin). Similarly, BE (at a concentration 10 *μ*M) demonstrated a marked decrease in viability of other murine melanoma, B164A5 cell line, resulting in a 52% reduction of viable cells compared to control [39], while it has moderate activity towards epidermoid carcinoma of the mouth KB cells (IC_50_ value >45.2 *μ*M) [38] and total inactivity towards melanoma SK-MEL2 cells with an IC_50_ value higher than 250 *μ*M [35]. Another skin cancer epidermoid carcinoma A431 cell line was much more sensitive to BE treatment; the concentrations 10 *μ*M (4.43 *μ*g/mL) and 30 *μ*M (13.28 *μ*g/mL) have inhibited proliferation by 63.42% and 70.30%, respectively [16], and the IC_50_ value was 6.76 *μ*M [32].

Cytotoxicity and antiproliferative activity of BE have also been confirmed towards a panel of human and murine haematological malignancies* in vitro*. BE has significantly suppressed cells growth in several models of leukaemia, HL60 and U937 cell lines [25], with the comparable IC_50_ values 14.7 and 14.4 *μ*M, respectively, but the most pronounced effect has been observed in Jurkat E6.1 cells—6.7 *μ*M [23]. Nearly two-fold weaker activity of BE towards human leukaemia CCRF/CEM cells versus mouse leukaemia P388 cell line has been observed (IC50 24.6 versus 12.4 *µ*M) [33]. Although this results have been contested by other studies that show a total lack of BE activity against CEM cells—IC_50_ value >250 *μ*M [35, 40, 41]. Similar discrepancies have been demonstrated towards human chronic myelogenous leukaemia K562 whereas on one hand BE is reported as active, IC_50_ value 14.5 *μ*M [25], while on the other hand it is completely inactive, IC_50_ values >200 *μ*M [26] and 250 *μ*M [35]. Additional studies have evidenced notable activity of BE in human multiple myeloma RPMI 8226 cell line, where the concentration 6.4 *μ*M inhibited growth of 50% cells after 48 h treatment [23].

The significant discrepancies between IC_50_ doses of BE towards the same cell lines, A549 [23, 26–29], T47D [23, 33], PC-3 [26–28, 35], CCRF/CEM [33, 35, 40, 41], and K562 [25, 26, 35], evaluated by different authors seem to be the result of various sources of BE and extraction procedures as well as lack of standardised treatment modalities (treatment times, doses, and individual features of each laboratory cell strains).

Conspicuously, BE shows antiproliferative and cytotoxic activity towards cancer cell lines resistant to conventional cytostatic drugs, which suggests a novel mechanism of action. BE has been shown to elicit significantly stronger antiproliferative effect (by means of IC_50_) values on the daunorubicin- and mitoxantrone-resistant cancer cells, such as the DB-resistant human gastric cancer 257RDB cell line (IC_50_ 10.97 *μ*M), and NOV-resistant (Novantrone) human gastric cancer 257RNOV cell line (IC_50_ 12.25 *μ*M), and human pancreatic carcinoma 181RNOV cell lines (IC_50_ 20.62 *μ*M) than on the drug-sensitive parental 257P and 181P cells [36], whereas BE has been inactive towards K562-Tax (paclitaxel-resistant subline of human chronic myelogenous leukaemia), with the IC50 value >250 *µ*M [35]. Nevertheless, BE has been suggested to overcome some forms of drug resistance in cancer cells refractory to conventional chemotherapeutic agents [36].

The purity and purification methods play important roles in the downstream activity of BE and its derivatives. A growing body of evidence suggests that different BE extracts have better therapeutic potential than pure BE. In some cases, isolated BE has been found to elicit a weaker antiproliferative activity against the human gastric cell line (EPG85-257P) (Table 1) as compared with a crude birch bark extract, while in other cases, stronger inhibitory effect towards pancreatic carcinoma cells (EPP85-181P) by isolated BE as compared to the birch bark extract has been observed [36]. The outer bark of the birch trees contains BE as the main component but some other pentacyclic triterpenes as well [42]. Thereby, the synergistic effects of combination of various triterpenes with diverse activities and modes of action could explain to some extent the discrepancies in results obtained* in vitro* between birch bark extract and purified BE. Although this action, or combination of actions, is cell type-dependent, for example, a crude birch bark extract (*B. pendula* Roth, syn.* B. verrucosa*-European White Birch) has been found to elicit more pronounced antiproliferative potential against the daunorubicin- and mitoxantrone-resistant human gastric and pancreatic carcinoma cell lines (IC_50_ values 4.29–7.08 *μ*M and 9.07–23.03 *μ*M, resp.), compared to the drug-sensitive parental 257P and 181P cell lines [36]. Likewise, the BE-enriched (approximately 97%) birch bark extract (*B. pendula* Roth) has shown strong antiproliferative potential towards human cancer cell lines A431, A2780, HeLa, and MCF7* in vitro*, with the IC_50_ values from 2.26 *μ*M up to 11.29 *μ*M (1 and 5 *μ*g/mL) [43]. In another study, bark extract from* B. pendula* Roth with content of 57.01% of BE at the concentration of 17.53 *μ*M (7.76 *μ*g/mL) and 52.61 *μ*M (23.29 *μ*g/mL) has inhibited proliferation of A431 (by 70.02% and 78.70%, resp.), MCF-7 (by 45.54% and 55.55%, resp.), and HeLa (by 70.62% and 76.23%, resp.) cells stronger than pure BE [16]. A highly purified triterpene extract (TE) from the* Betulae* cortex with BE as a main component (up to 87.3% w/w of identified triterpenes) demonstrated a dose-dependent cytotoxicity from 0.090 *μ*M (0.04 *μ*g/mL) to 90.35 *μ*M (40 *μ*g/mL) in human nonmalignant, immortalized keratinocytes (HaCaT) and skin cancer A431 (squamous cell carcinoma) cell lines, similar to its main constituents, BE and betulinic acid (BA). TE has been shown to form an oleogel, which facilitates an application on the skin for dermatological indications [44].

An essential advantage of the use of BE as bioactive agent is its relatively low toxicity towards noncancerous cells [45]. BE has shown relatively modest cytotoxicity against human skin fibroblasts (HSF)—doses below 10 *μ*M have no apparent toxicity [23]—and mouse fibroblasts (Balb3T3)—IC_50_ value 106.8 *μ*M (47.3 *μ*g/mL) [33]. Also, BE has expressed low activity towards immortalized human epithelial cells (hTERT-RPE1cell line) and human umbilical vein endothelial cells (HUVEC) with the IC_50_ values >45 *μ*M (20 *μ*g/mL) [19]. BE isolated from the tropical plant* Cyrtomium fortumei* (J.) or BE from the roots of* Belamcanda chinensis* (L.) DC inhibited the growth of NIH3T3 mouse fibroblasts only by 29.8% and 33.5%, respectively, at a concentration 20 *μ*M [20, 22].

On the other hand, BE has shown significant antiproliferative effect against human normal skin fibroblasts (WS1), with the IC_50_ value 3.6 *μ*M [27, 28] and normal lung fibroblasts WI38 (IC_50_ 15.2 *μ*M) [25]. Although there are only few reports concerning BE influence on normal cells, noncancerous cells of various origins have been confirmed to be more resistant to BE treatment than tumour cells pointing to some cell-type selectivity. These encouraging results of* in vitro* studies make BE a promising therapeutic candidate.

BE has been shown to markedly impede the migration of several cancer cell types, including lung (lung carcinoma A549 cells) and central nervous system tumours (cell lines C6—glioma and TE671—rhabdomyosarcoma/medulloblastoma) [23].

*In vivo* antiangiogenic effects have also been reported for BE. Using the chorioallantoic membrane (CAM) model in chicken embryos, to study blood vessel formation, the antiangiogenic activity of BE has been proved by inhibition of the formation of new capillaries, presumably throughout targeting the endothelial cells [43]. This activity can be further enhanced by using BE in nanoemulsion formulation to increase penetrability to extraembryonic tissues [46]. Similarly, the decrease in melanoma tumour size in C57BL/6J mice model (at post-B164A5 tumour cells inoculation) after BE treatment has been attributed to its antiangiogenic activity. Indeed, immunocytochemical analyses showed a reduced VEGF expression in mice treated with BE-*γ*-cyclodextrin derivative (GCDG) complex in comparison with the control group [39]. The molecular basis of BE antimigration and antiangiogenic activities remains to be determined.

## 3. Potential Mechanisms of BE-Mediated Anticancer Activity

A rapidly rising number of studies have shown that the induction of apoptotic cell death is an essential mechanism of anticancer agents activity [47–49], including BE. It has been demonstrated that disruption of the apoptosis machinery is a typical feature of tumour cells [50–52]. Apoptosis is a type of programmed cell death, characterized by a series of complex, specific biochemical and cytomorphological events. Two main pathways of apoptosis have been identified, the extrinsic (death receptor-related) and the intrinsic (mitochondrion-dependent). The extrinsic pathway is initiated by external signals, for instance, the binding of molecules (ligands), such as Fas, TNF, or TRAIL, to their respective death receptors, localized in the cell surface. The intrinsic apoptosis pathway is activated by different stimuli, such as DNA damages, oxidative stress, radiation, and growth factors withdrawal [53].

An ability to trigger apoptosis in tumour cells has been proved as one of mechanisms underlying BE cytotoxicity and its antiproliferative potential. BE treatment has resulted in cytomorphological alterations characteristic for cells undergoing apoptosis, like cell rounding, chromatin condensation, nuclear fragmentation, membrane blebbing, and formation of apoptotic bodies [26]. Likewise, inhibition of HeLa cells proliferation has been accompanied by morphological changes, characteristic of apoptosis: cells have become smaller and the morphology has showed karyopycnosis, when exposed to BE for 24 h, and the effect was a dose-dependent [24]. BE treatment of murine melanoma cells B164A5 has demonstrated almost equal amounts of apoptotic and dead (necrotic) cells [39]. BE has been shown to induce apoptotic cell death in human lung adenocarcinoma cells* in vitro* (A549 cell line). The amount of apoptotic cells has significantly increased by 27.64% in comparison with control, untreated cells [29]. BE has been shown to increase substantially the number of cytosolic oligonucleosomal fragments in A549 cell line [23]. More detailed studies have shown that BE induces apoptosis of human cancer cells through the mitochondrial (intrinsic) pathway in A549, Jurkat [54] and HeLa cancer cell lines [26, 54]. BE proapoptotic activity in HeLa cells has involved the sequential activation of caspases 9, 3, and 7 and the cleavage of poly (ADP-ribose) polymerase (PARP) [24]. The cleavage of caspase-3 substrate PARP to the 85 kDa form of the protein has been observed, which points at a caspase-activated apoptotic cell death. The activity of caspase-8 remained unchanged, suggesting a lack of extrinsic pathway activation, while caspase-9 has been shown to be initially activated, followed by cytochrome c/Smac proteins release from the mitochondrial intermembrane space, mitochondrial membrane potential depolarization, and rapid translocation to the mitochondrion of Bax and Bak proteins (proapoptotic members of the Bcl-2 family) [26]. In another study, BE had no influence on the total expression of Bax and Bcl-2, on mRNA as well as on protein level, and the total expression of Bak protein in HT-29 cancer cells [23]. However, a few reports have demonstrated that BE treatment induced the expression of other cellular proteins indirectly involved in apoptosis. By means of pharmacoproteomic approach, BE has been shown to upregulate aconitate hydratase and malate dehydrogenase in cancer cells, enzymes involved in ATP generation, supporting the involvement of mitochondrial pathway as the main mechanisms of BE-induced apoptotic cell death [29]. BE-mediated downregulation of isoform 1 of 3-hydroxyacyl-CoA dehydrogenase type 2, also known as enoyl-CoA hydratase, an enzyme related to lipid metabolism, should be further investigated to elucidate its involvement in BE-induced apoptosis. BE treatment resulted also in decrease of poly (rC)-binding protein 1 expression. The poly (rC)-binding protein 1 was reported to protect cells from different apoptosis inducers and modulate heat shock protein 90-*α* 2 (HSP90-*α* 2) expression, which is involved in the regulation of mitochondrial membrane permeabilization and cytochrome c release. This might be a mechanism by which BE sensitises cancer cells to undergo apoptosis. Moreover, a highly purified TE from* Betulae* cortex, containing BE as a main component, displays a dose-dependent proapoptotic effects on HaCaT and A431 cells, similar to its main constituents, BE and BA [44].

Apoptosis induction is often a consequence of cell cycle disturbances. The cell cycle progression is controlled by cyclins, which are a regulatory proteins family of cell cycle-dependent kinases (CDKs) [55]. Regulation of the cell cycle has become a challenge and a promising target for cancer therapy [56]. Thus, numerous anticancer agents have been reported to arrest cell cycle at the G_0_/G_1_, S, or G_2_/M phases and consequently trigger apoptosis of cancer cells [57–60].

Surprisingly, limited attention has been given to the regulation of cell cycle by BE in cancer cells. BE at a concentration 10 *μ*M has been shown to induce an arrest of murine melanoma B164A5 cells in S phase, with a concomitant decrease in the number of cells in the G_0_/G_1_ phases [39]. BE treatment of HepG2 cells (hepatoma) induced a late stage G_0_/G_1_ phase cell cycle arrest, and at the early stage S phase, and a subsequent decrease in the amount of cells in the G_2_/M phases at a relatively low concentration (11.29 *μ*M/5 *μ*g/mL). Another study, using hepatoma Hep3B cells, has shown that BE treatment resulted in a cell cycle arrest at the G_2_/M phase, showing different effects of BE in regulation of the cell cycle, depending on hepatoma cells type. Furthermore, BE has been reported to slightly reduce DNA replication, without influencing the expression level of cell cycle regulatory genes, p21 and p53 in hepatoma cells [61]. p21 and p53 expression level were also not affected after BE treatment in other tumor cell lines originating from central nervous system (medulloblastoma/rhabdomyosarcoma, neuroblastoma, and glioma) and various peripheral cancers including lung, colon, thyroid, breast, leukaemia, multiple myeloma, and several tumour primary cultures [23].

Cell division perturbations after BE treatment could be linked to direct interactions with DNA topoisomerases (Topo), but not with DNA, at concentrations comparable with those of the well-known inhibitor etoposide. BE, among other lupane- and oleanane-type triterpenoids from the bark of* Phyllanthus flexuosus*, has been reported to selectively inhibit the activity of human Topo II in a dose-dependent manner. Topo are known to play an essential role in DNA metabolism, affecting replication, transcription, recombination, and mitotic chromosome segregation [62]. Thereby, Topo might be a target for the antitumour activity of BE. Topo I inhibitors are known to induce apoptosis in cancer cells [63, 64]. Whereas BE affects Topo II activity, it has no influence on the activity of human Topo I [25].

Another enzyme involved in cell division and affected by BE treatment (IC_50_ 20 *μ*M) is cAK (cyclic AMP-dependent protein kinase) which is activated by a plethora of extra- and intracellular signals. A central network player, cAK, is involved in the regulation of a variety of cellular processes including metabolism, cell division, specific gene expression, and development [65]. The inhibition of cAK by BE is specific as no changes in the activity of ERK1/2 and AKT kinases were observed [23]; the two latter kinases are frequently pathologically hyper-activated in several human cancers [66, 67].

BE has been searched for its effect on human melanocortin (MC) receptor signalling pathway. Human MC receptors-expressing COS-7 cells bind BE with different specificities depending on the MC subtype. The affinity of BE to the MCRs is MC1>MC3>MC5>MC4. Furthermore, BE antagonizes *α*-melanocyte-stimulating hormone- (*α*-MSH-) induced accumulation of cAMP to some extent in the mouse melanoma cell line B16-F1, which naturally expresses MC1 receptor without stimulating MC receptor-associated generation of cAMP [68]. MC1 receptor subtype is expressed almost in each cutaneous cell type, in immune and in melanoma cells [69, 70]. It is also worth mentioning that the MC1 receptor has been suggested to be a crucial modulator of epidermal melanocyte proliferation and differentiation [71, 72] and has been suggested as an important target of the antimelanoma activity of BE and its structurally similar substances, such as BA [68].

## 4. Inhibition of Carcinogenesis and Antimutagenic Activity* In Vivo*

BE has been confirmed as a potent antimutagenic agent of skin carcinogenesis. The topical formulation with BE nanoemulsion has been tested on C57BL/6J type mouse skin, chemically damaged by DMBA (7,12-dimethylbenz[*α*]anthracene) as a tumour initiator and 12-O-tetradecanoylphorbol-13-acetate (TPA) as tumour promoter. Potentially, any damage of the skin surface might lead to significant pathologies, such as skin neoplasms. Observations of cutaneous damages have revealed the activity of BE in reducing skin lesions and irritation by considerably decreasing erythema [73]. Topical application of BE has exhibited distant effects and influenced the respiratory function of isolated liver mitochondria in a two-stage model of skin carcinoma induced in mice. The improvement of liver mitochondrial respiration and increased basal (LEAK state) and active (OXPHOS state) respiration has been observed. Moreover, BE may also influence the penetration of carcinogens and reduce damage in main organs, such as liver, since application of carcinogens on the skin surface, because of their slow penetration, leads to toxic effects especially on liver. BE has also been shown to inhibit apparition and promotion of skin tumours [46]. Similarly, birch bark dry extract (BDE, with BE as a main component—at least 70%) has been applied on mice with chemically-induced mutagenesis. The administration of 150 and 1500 mg/kg BDE to mice resulted in no mutagenic and comutagenic effects. The number of cells with chromosomal aberrations was comparable between control and BDE-treated animals. Furthermore, BDE in doses of 50, 150, and 450 mg/kg notably reduced the cytogenetic effect of mutagens, dioxidine (1,4-di-N-oxide of 2,3-bis-(hydroxymethyl) quinoxaline, DN) and cyclophosphamide (N′-bis-(b-chloroethyl)-N′-O-trimethyl ester of phosphoric acid diamine, CP). A single treatment with BDE in doses of 50 and 150 mg/kg results in approximately the same antimutagenic effect and decreased the damaging activity of DN and CP by 53–60% and 60%, respectively. BDE inhibits free radical oxidation and thus the prooxidant mutagenic activity of DN. The protective activity of BDE has been potentially mediated by various mechanisms, for instance,* via* inhibition of cytochromes P450, playing a crucial role in the metabolism of CP, or by stimulation of production of interferons, which may improve DNA repair [74].

## 5. Potential Application in Therapy

No typical clinical trials have been published using BE for the treatment of human cancer so far [12]. Nevertheless, a nonrandomized pilot study, using a birch bark extract to treat actinic keratoses (AK) [75, 76], suggests a preventive and therapeutic potency of BE in skin pathologies supporting by encouraging* in vivo* studies [73]. AK is considered to represent an early and noninvasive squamous cell carcinoma* in situ*, due to histological similarity [77], and as commonly diagnosed skin damage induced by ultraviolet light should be treated to avoid the development of nonmelanoma skin cancers [78]. A birch bark ointment (containing around 87% of the triterpenes with predominant content of BE, 80%), used as monotherapy for the treatment of AK, resulted in a remission of more than 75% of the lesions in 79% of the patients after treatment as a product that has been approved for use as a cosmetic in Germany [75]. Furthermore, recent tests with water-free BE-based oleogel containing a higher extract concentration have confirmed the effectiveness of the BE-based strategy in the therapy of AK. The treatment resulted in complete clearing of the lesions in 64% and partial remission (more than 75% of lesions) in 86% of the patients, after a three-month treatment period, comparably to standard therapy (cryotherapy) [76]. Additionally, a synergistic effect by the combination of BE and cryotherapy has been reported with no observable undesirable effects [75]. Besides, BE-based oleogel decreased the degree of epidermal dysplasia and number of dyskeratoses in treated patients during a prospective, randomized, and comparative clinical phase 2a study. Excellent skin tolerance for oleogel prepared from a standardized triterpene dry birch bark extract was also noticed [76]. For that reason, the treatment with birch bark ointment or BE-based oleogel is regarded as a new topical alternative for current AK therapy and a promising chemopreventive agent, especially that the risk of AK progression to invasive type of squamous cell carcinoma has been estimated between 1% and 16% [79].

In animal models and pilot studies with BE, BE-based oleogel, or triterpene birch bark extract, no severe adverse effects have been observed. BE, likewise other pentacyclic triterpenes, has also shown no toxicity. Daily administration of BE (doses at 540 mg/kg of body weight i.p. in rats and 300 mg/kg s.c. in dogs) resulted in very low toxicity, if any [42]. Thereby, it seems that triterpene birch bark extract and its representative compound, BE, are safe to use* in vivo*.

## 6. Concluding Remarks

An increasing number of studies support the antineoplastic activity of BE. A limitation for TE's biological and pharmacological effectiveness is their poor solubility. The solution could be a complexation with hydrophilic carriers. Indeed, BE hydrosolubility can be significantly improved by highly hydrophilic semisynthetic *β*-cyclodextrin [80], and *γ*-cyclodextrin derivatives [39] as carriers, which has enhanced antiproliferative potential of BE towards cancer cell lines [80], and by incorporation in nanoemulsion [46], which may increase its bioavailability and consequently improve its activity* in vitro* and* in vivo*. Chemically synthesized cyclodextrin derivatives offer the prospect of preparation highly stable complexes with both BE and other terpenes, such as BA [81], and possibly might be submitted for clinical trials soon. Likewise, application of cholesterol containing BE-liposomes may be considered as a promising method to facilitate the use of BE in the context of anticancer therapy [54].

Due to the multitarget activity of BE on cancer cells, it may be used in combination with commonly used chemotherapeutic drugs, as their synergistic effect can help to eliminate cancer cells, including drug-resistant cells [36]. Another novel approach for the application of BE in cancer therapy may be its chemical modification with various ligands which allows obtaining an enhanced cytotoxicity towards tumour cells, better solubility, and bioavailability than the parental compound [33]. Therefore, BE has been attempted to be used as a precursor in the synthesis of novel BE derivatives with improved anticancer and pharmacokinetic properties.

Many of the molecular mechanisms of action of TE are still elusive which limits our understanding of this potentially beneficial group of natural compounds.

## Figures and Tables

**Table 1:** *In vitro* antiproliferative effect of BE on human and animal cancer cell lines by means of IC_50_ values (inhibitory concentration 50%).

Cancer type	Cell line	IC_50_		References
		*μ*M	*μ*g/mL	
Human myelogenous leukaemia	K562	**14.5**	6.4	[25]
		>225.9	**>100.0**	[26]
		**>250.0**	>111.0	[35]

Human neuroblastoma	SK-N-AS	**2.5**	1.1	[23]
Human rhabdomyosarcoma/medulloblastoma	TE671	**10.3**	4.6	

Human neuroblastoma	GOTO	**17.1**	7.6	[25]
Human neuroblastoma	NB-1	**16.5**	7.3	

Rat glioma	C6	**5.9**	2.6	[23]
Human thyroid carcinoma	FTC 238	**6.8**	3.0	

Human lung cancer	Lu1	>45.2	**>20.0**	[19]

Human nonsmall cell lung carcinoma	NCI-H460	63.5	**28.1**	[26]

Human lung carcinoma	A549	**20.0**	8.9	[29]
		33.4	**14.8**	[26]
		**7.4**	3.3	[23]
		**3.8**	1.7	[27, 28]
Human breast adenocarcinoma	MCF-7	**23.3**	10.3	
		30.7	**13.6**	[26]
		**8.32**	3.7	[32]

Human breast carcinoma	T47D	**5.2**	2.3	[23]
		**73.2**	32.4	[33]

Human cervical carcinoma	HeLa	74.1	**32.8**	24 h [24]
		57.1	**25.3**	48 h [24]
		34.4	**15.2**	72 h [24]
		22.6	**10.0**	[26]
		**6.7**	2.9	[32]

Human ovarian carcinoma cells	A2780	>45.2	**>20.0**	[21]

Human prostate adenocarcinoma	PC-3	**17.9**	7.9	[27, 28]
		82.9	**36.7**	[26]

Hormone-dependent human prostate cancer	LNCaP	>45.2	**>20.0**	[19]

Human gastric carcinoma	EPG85-257P	**18.7**	8.3	[36]
Human pancreatic carcinoma	EPP85-181P	**21.1**	9.3	

Human colorectal adenocarcinoma	DLD-1	**6.6**	2.9	[27, 28]

Human colorectal adenocarcinoma	HT-29	**4.3**	1.9	[23]

Human colon cancer	Col2	45.2	**>20.0**	[19]

Human colorectal adenocarcinoma	SW707	**51.7**	22.9	[33]

Human hepatoma	HepG2	22.8	**10.1**	[26]
Human hepatocarcinoma	SK-HEP-1	132.1	**58.5**	

Human melanoma	G361	**12.4**	5.5	[25]
Human melanoma	SK-MEL-28	**16.2**	7.2	

Mouse melanoma	B16-F1	**13.8**	6.1	[27]

Mouse melanoma	B16 2F2	**27.4**	12.1	[37]

Human melanoma	MEL-2	>45.2	**>20.0**	[38]

Human melanoma	SK-MEL2	**>250.0**	>111.0	[35]

Human skin epidermoid carcinoma	A431	**6.8**	3.0	[32]

Human promyeloblastic leukaemia	HL60	**14.7**	6.5	[25]
Human leukaemia	U937	**14.4**	6.4	

Human T lymphoblast leukaemia	Jurkat E6.1	**6.7**	3.0	[23]

Mouse leukaemia	P388	**12.4**	5.5	[33]
Human leukaemia	CCRF/CEM	**24.6**	10.9	

Human multiple myeloma	RPMI 8226	**6.4**	2.8	[23]

Human oral epidermoid carcinoma	KB	>45.2	**>20.0**	[19]

Gastric carcinoma, atypical mitoxantrone MDR variant	EPG85-257RNOV	**12.3**	5.4	[36]
Gastric carcinoma, classical daunorubicin MDR variant	EPG85-257RDB	**11.0**	4.9	
Pancreatic carcinoma, atypical mitoxantrone MDR variant	EPP85-181RNOV	**20.6**	9.1	
Pancreatic carcinoma, classical daunorubicin MDR variant	EPP85-181RDB	**26.5**	11.7	

Human myelogenous leukaemia (paclitaxel-resistant)	K562-Tax	**250.0**	111.0	[35]

To facilitate comparison, the doses were recalculated to *μ*M or *μ*g/mL. Original data are presented in bold.

**Table 2:** *In vitro* antiproliferative effect of BE on human tumour primary cultures by means of IC_50_ values (inhibitory concentration 50%).

Tumour type	Primary culture	IC_50_		References
		*μ*M	*μ*g/mL	
Ovarian carcinoma	HPOC	**2.8**	1.2	[23]
Cervical carcinoma	HPCC	**3.4**	1.5	
Glioblastoma multiforme	HPGBM	**3.4**	1.5	

To facilitate comparison, the doses were recalculated to *μ*M or *μ*g/mL. Original data are presented in bold.
